# Harm Reduction and Treatment Among People at High Risk of Overdose

**DOI:** 10.1001/jamanetworkopen.2024.27241

**Published:** 2024-08-12

**Authors:** Sachini Bandara, Lauren Byrne, Vanessa Berman, Adrienne Hurst, Dionna King, Jason B. Gibbons, Olivia K. Sugarman, Amy Livingston, Lindsey Kerins, Eric G. Hulsey, Alejandro Alves, Brendan Saloner

**Affiliations:** 1 Department of Mental Health, Johns Hopkins Bloomberg School of Public Health, Baltimore, Maryland; 2 Department of Health Policy & Management, Johns Hopkins Bloomberg School of Public Health, Baltimore, Maryland; 3 Pacific Institute for Research and Evaluation, Chapel Hill, North Carolina; 4 Vital Strategies, New York, New York; 5 The University of Colorado Anschutz Medical Campus, Aurora

## Abstract

**Importance:**

Rates of overdose deaths involving synthetic opioids remain high, increasingly involve stimulants combined with opioids, and are increasing rapidly in racially and ethnically minoritized communities, yet little is known about access to harm reduction and treatment services in these groups.

**Objective:**

To characterize access and barriers to harm reduction and treatment in a racially and ethnically diverse population of people who use drugs.

**Design, Setting, and Participants:**

A cross-sectional telephone survey of people recruited from 39 treatment, harm reduction, and social service organizations in Milwaukee County, Wisconsin; Flint and Detroit, Michigan; and statewide in New Jersey was conducted from January 30 to July 28, 2023. Adults who used cocaine, methamphetamine, or opioids in the past 30 days called a study hotline and completed an interview in English or Spanish.

**Exposures:**

Overdose experience, drug types used (opioids only, stimulants only, and polysubstance), and social risk factors (eg, financial instability and criminal legal involvement).

**Main Outcomes and Measures:**

Recent use of any harm reduction services, fentanyl test strips, naloxone possession, treatment, and self-reported barriers to services.

**Results:**

Of the total sample of 1240 adults, 486 (39.2%) were Black non-Hispanic, 183 (14.8%) were Hispanic, and 464 (37.4%) were White non-Hispanic. In the past 30 days, 826 individuals (66.6%) were polysubstance users, 135 (10.9%) used only opioids, and 279 (22.5%) used only stimulants. A total of 349 respondents (28.1%) experienced a prior-year overdose. Compared with those without a prior-year overdose, people with overdose were more likely to possess naloxone (80.7% vs 68.2%; *P* < .001), possess fentanyl test strips (36.8% vs 23.5%; *P* < .001), and use harm reduction services (63.4% vs 53.0%; *P* = .003), while differences in treatment use were nonsignificant (52.0% vs 46.6%; *P* = .24). Among stimulant-only users, 51.4% possessed naloxone compared with 77.3% of opioid-only users (*P* < .001) and 77.6% of polysubstance users (*P* < .001), with similar disparities in fentanyl test strip possession.

**Conclusions and Relevance:**

In this cross-sectional study of people who used drugs in the past 30 days, findings highlighted low use of harm reduction and treatment services among people who use stimulants. Additional communication regarding their importance may help increase the use of the services amidst a rapidly changing drug supply.

## Key Points

**Question:**

Does access to treatment and harm reduction differ by overdose history and drug type in a racially and ethnically diverse population of people who use drugs?

**Findings:**

In this cross-sectional study of 1240 people who use drugs in 3 states, the proportion of respondents reporting use of fentanyl test strips was 36.8% among past year overdose survivors and 23.5% among those not experiencing an overdose—a significant difference. Approximately half (47.9%) of all participants received treatment in the past 30 days.

**Meaning:**

The findings of this study suggest there are substantial gaps in the use of evidence-based treatment and harm reduction services that could reduce overdose risk.

## Teaser

This cross-sectional study examines the use of treatment and harm reduction services among individuals who use drugs.

## Introduction

The US continues to grapple with a severe overdose crisis, with overdose deaths reaching more than 100 000 in 2022 and more than 1 million total lives lost since 1999.^1,2^ The crisis has evolved as the drug supply has changed, and opioid overdose deaths now overwhelmingly involve illicitly manufactured fentanyl.^2,3,4^There has also been a sharp increase in overdose deaths involving cocaine and methamphetamine, and other toxic adulterants, such as xylazine, have proliferated in the drug supply.^5,6^ Drug overdose mortality has increased most rapidly among non-Hispanic Black populations and older populations.^2,7^

Despite the evolving changes in the illicit drug supply and the changing demographic characteristics of overdose decedents, little is known about access to harm reduction and treatment services among people at the highest risk of fatal overdose. The National Survey on Drug Use in Health (NSDUH) provides estimates of drug use and treatment use in the general population, but the data are released with a 2-year time lag, reducing its value for timely intervention. Moreover, the NSDUH likely underestimates certain types of substance use (eg, injection drugs) and the prevalence of specific substance use disorders and has limited coverage of key populations at the highest risk for overdose, such as those who are housing insecure.^8,9,10,11^ The NSDUH does not include estimates of nonfatal overdose and key behavioral and service-related factors, such as the use of harm reduction programs and access to fentanyl test strips and naloxone. Insights into evolving changes in service use also come from surveys of people who use drugs, which report that the use of treatment and harm reduction services, such as syringe service programs, fentanyl test strips, and naloxone, is associated with the type and frequency of drug use, polysubstance use, race and ethnicity, overdose experience, and other structural vulnerabilities.^4,12,13,14,15,16,17,18,19,20^ However, many of these surveys are small in scale, limited to clients of a small number of service providers, limited to certain types of drug use (eg, opioids), and predominately comprise younger, non-Hispanic White respondents. Studies of people who use stimulants in the era of fentanyl contamination find barriers to accessing and using fentanyl test strips, harm reduction services, and treatment.^4,21,22,23,24,25,26^ Many of these studies provide rich qualitative data, but further triangulation with larger scale surveillance and survey data is needed.

In response to the need for rapid surveillance of overdose risk factors, we fielded the VOICES survey in 2023 as part of a public health campaign to reduce overdose deaths, both generally and among Black and Hispanic people who use drugs. The purpose of the study was to collect data directly from people who use drugs to learn about their experiences and attitudes toward drug use, harm reduction, treatment, overdose, and general health and wellness. VOICES had high representation of key groups experiencing increasing rates of overdose, including people who use stimulants with opioids, Black and Hispanic people, and older adults. Motivated by literature examining high barriers to accessing harm reduction and treatment services among these key populations,^12,13,14,15,16,17,18,19,20^ we examined the use of harm reduction and treatment services, recent experience of overdose, and individual risk factors in a large multistate population of people who use drugs who are at high risk for overdose, and further describe self-identified barriers to using services that reduce overdose risk.

## Methods

VOICES was an anonymous telephone survey of a targeted, nonprobability sample of adults at high risk for overdose who use drugs in Wisconsin, New Jersey, and Michigan. This study was approved by the Johns Hopkins Bloomberg School of Public Health Institutional Review Board and follows the Strengthening the Reporting of Observational Studies in Epidemiology (STROBE) reporting guideline. Participants provided oral consent and received a $25 gift card incentive.

### Sample

The survey included adults who had used drugs from Milwaukee County, Wisconsin; Flint and Detroit, Michigan; and throughout New Jersey. These locations are part of the Bloomberg Overdose Prevention Initiative, a multistate effort to reduce overdose deaths that supports interventions provided through government and community-based organizations. Survey results informed technical assistance to partnered community organizations.

Participants were recruited from 39 community-based sites. Sites included harm reduction, treatment, general medical services, and social service professionals (eTable 1 in Supplement 1). Sites were provided with a $2000 honorarium for participation and allocated a limited number of recruitment cards based on the size of their clientele to distribute to clients via office-based settings and mobile/street outreach teams. Sites were chosen because they served a population at risk for overdose, clients in zip codes with a high percentage of Black and/or Hispanic residents, and to balance the percentage of the sample that was treatment engaged. To improve data collection from those not engaged with a service organization, a portion of individuals were given peer recruitment cards that they could distribute to peers who may also be eligible for the survey but were not engaged in services. Eligible survey respondents included those who were at least aged 18 years; had used crack, cocaine, opioids, or methamphetamines in the past year; and had the cognitive ability to consent to participate and complete the survey.

The focus of this analysis was those at highest risk for overdose. Therefore, we limited the sample in this analysis to individuals who used opioids and/or stimulants in the past 30 days (N = 1240).

### Data Collection

Each recruitment card contained a unique survey ID and telephone number. Individuals could call the survey telephone line, provide the recruitment ID, and, if eligible, complete a 30-minute anonymous survey. The telephone line was staffed with both English- and Spanish-speaking data collectors and was operational at least 40 hours a week, including on nights and weekends, to coincide with when sites were recruiting clients. Telephone line operators collected survey responses from January 30 through July 28, 2023.

### Survey Instrument

The survey instrument included 75 questions covering the following domains: current drug use behaviors, overdose experiences, perceptions of overdose risk, harm reduction use, substance use disorder treatment use, structural vulnerabilities (eg, housing and food insecurity), general health, and self-reported sociodemographic characteristics (eMethods in Supplement 1). Study protocols were informed by a community advisory board consisting of 8 individuals who represented individuals with lived experience, recruitment sites, and state agencies responsible for overdose prevention policy.

### Measures

Sociodemographic measures in this study included age (18-29, 30-39, 40-49, 50-59, and ≥60 years), race and ethnicity (Black non-Hispanic, Hispanic, White non-Hispanic, and Other non-Hispanic), gender (female, male, and other), employment status (employed parttime or full time, unemployed for health or nonhealth reasons, and other, including retired), and self-reported health (dichotomized as excellent, very good, or good vs fair or poor).

We included 3 measures of structural vulnerabilities in this analysis: housing instability, financial insecurity, and criminal-legal system involvement. We measured housing instability (“Are you worried or concerned that in the next year you may not have stable housing that you own, rent, or stay in as a part of a household?”), financial insecurity (“In the past year, has it been difficult for you to pay for your basic needs, like food, housing, or other bills?”), and criminal-legal involvement as self-reported time spent in jail, prison, or on probation, parole, supervised release, or other conditional release in the past year.

Drug use characteristics in this study included the type of drugs used, mode of drug use, and frequency of drug use in the past 30 days. We asked respondents about use of opioids, stimulants, and tranquilizers, benzodiazepines and limited the analysis to respondents who used opioids and/or stimulants in the past 30 days. We categorized drug type as use of only opioids (heroin, fentanyl, opioid analgesics, or unprescribed buprenorphine or methadone), use of only stimulants (crack or cocaine, methamphetamine, speed, or other stimulants), or polysubstance use (use of at least 2 types of drugs: opioids, stimulants, and tranquilizers or benzodiazepines). Modes of drug use were not mutually exclusive and included injecting, smoking, snorting, swallowing, or other modes. We categorized the frequency of drug use as more than once a day, once a day, a few times a week, a few times a month, or only once.

Other measures included past year overdose experience, past month substance use disorder treatment receipt (defined as receiving treatment, medication, or counseling from a medical professional or professional counselor, not including peer-led groups), past month harm reduction service use (defined as services providing safer drug use supplies, such as naloxone, fentanyl test strips, sterile needles, and safer smoking equipment), past month use of fentanyl test strips, and currently possessing a naloxone kit.

To assess barriers to accessing treatment, we asked all survey respondents if they had ever wanted to receive treatment but could not get it. To those responding yes, we included a follow-up question asking why not. Similar questions were used for harm reduction services and use of fentanyl test strips. Data collectors coded the open-ended responses for all 3 questions into non–mutually exclusive prespecified categories.

### Statistical Analysis

We first identified variables of nonfatal overdose experience by estimating unadjusted and adjusted logistic regression models with sociodemographic characteristics (age, sex, and race and ethnicity), structural vulnerability (food insecurity, lack of housing, and criminal legal involvement), drug use in the past 30 days (type of drugs used and route of administration), and recruitment state as individual variables. We then examined the association between overdose experience, drug type, drug use frequency, mode of drug use, food insecurity, lack of housing, criminal legal involvement, and 4 outcomes: (1) past 30-day substance use treatment service use, (2) past 30-day harm reduction service use, (3) past 30-day fentanyl test strip use, and (4) whether they currently had naloxone. We estimated separate logistic regression models for each outcome and predictor adjusting by age, sex, and race and ethnicity. We clustered SEs by recruitment site and used 2-sided, unpaired Wald tests to identify differences between categories, applying *P* < .05 as a threshold for statistical significance. In addition, to describe potential barriers to substance use treatment and harm reduction services, we separately calculated the prevalence of each barrier to accessing treatment services, harm reduction services, and fentanyl test strips. Data were analyzed using Stata, version 18 (StataCorp LLC) and SAS, version 9.4 (SAS Institute Inc).

## Results

Of the total sample of 1240 adults, 344 (27.7%) were recruited from Detroit and Flint, Michigan; 499 (40.2%) from New Jersey; and 397 (32.0%) from Milwaukee County, Wisconsin (Table 1). Eight percent (n = 102) of the sample was recruited via peers. Most of the sample were older than 40 years, with 10.3% aged 18 to 29 years; 29.0%, 30 to 39 years; 22.5%, 40 to 49 years; 27.5%, 50 to 59 years; and 10.6%, aged 60 years or older (Table 1). The sample self-reported as 55.2% male and 43.2% female; 39.2% Black non-Hispanic, 14.8% Hispanic, and 37.4% White non-Hispanic, and 7.4% Alaskan Native, American Indian, Asian, multiracial, Native American, or Other. Only 18 individuals in the sample identified as non-Hispanic Native American or Alaska Native. The sample faced major challenges and structural vulnerabilities, with 71.3% being unemployed, 46.4% self-reporting poor or fair health, 54.4% experiencing housing instability, 73.6% experiencing financial insecurity, and 26.9% having recent criminal legal involvement. In the past 30 days, 47.0% of the sample used drugs more than once a day. In the past 30 days, 66.6% of the sample were polysubstance users, 10.9% used only opioids, and 22.5% used only stimulants. Among the polysubstance users, 53.4% reported using opioids and stimulants; 36.1% reported using opioids, stimulants, and tranquilizers/benzodiazepines; 6.8% reported using opioids and tranquilizers/benzodiazepines; and 3.8% reported using stimulants and tranquilizers/benzodiazepines.

**Table 1.** Sample Demographic Characteristics and Structural Vulnerabilities in 1240 Individuals

Variable	Participants, No. (%)
State	
Michigan	344 (27.7)
New Jersey	499 (40.2)
Wisconsin	397 (32.0)
Recruited from peer	102 (8.0)
Age range, y	
18-29	128 (10.3)
30-39	360 (29.0)
40-49	279 (22.5)
50-59	341 (27.5)
≥60	132 (10.6)
Race and ethnicity	
Black non-Hispanic	486 (39.2)
Hispanic	183 (14.8)
White non-Hispanic	464 (37.4)
Other^a^	92 (7.4)
Missing	15 (1.2)
Gender	
Female	536 (43.2)
Male	685 (55.2)
Other	10 (0.8)
Missing	9 (0.7)
Parent or guardian	253 (20.4)
Employment	
Employed	260 (21.0)
Unemployed	884 (71.3)
Other	87 (7.0)
Missing	9 (0.7)
Self-reported poor or fair health	575 (46.4)
Housing instability	674 (54.4)
Financial insecurity	913 (73.6)
Criminal legal involvement	333 (26.9)
Drug type	
Opioids only	135 (10.9)
Stimulants only	279 (22.5)
Polysubstance use	826 (66.6)
Mode of drug use^b^	
Injection	402 (32.4)
Smoke	821 (66.2)
Snort	603 (48.6)
Swallow	287 (23.1)
Other	11 (0.9)
Past 30-d drug use frequency	
More than once a day	583 (47.0)
Once a day	128 (10.3)
A few times a week	351 (28.3)
A few times a month	147 (11.9)
Only once	31 (2.5)
Past year overdose	349 (28.1)
Past 30-d substance use treatment	594 (47.9)
Past 30-d harm reduction service	684 (55.2)
Past 30-d fentanyl test strip	338 (27.3)
Currently has naloxone kit	882 (71.1)

^a^ Included respondents identifying as Alaskan Native, American Indian, Asian, multiracial, Native American, or Other.

^b^ Modes of drug use were not mutually exclusive.

Over a quarter (349 [28.1%]) of the sample had experienced an overdose in the past year. In the past 30 days, 47.9% of the sample received some substance use disorder treatment, 55.2% used harm reduction services, and only 27.3% used fentanyl test strips. Almost three-quarters (71.1%) currently possessed a naloxone kit.

**Figure 1.**
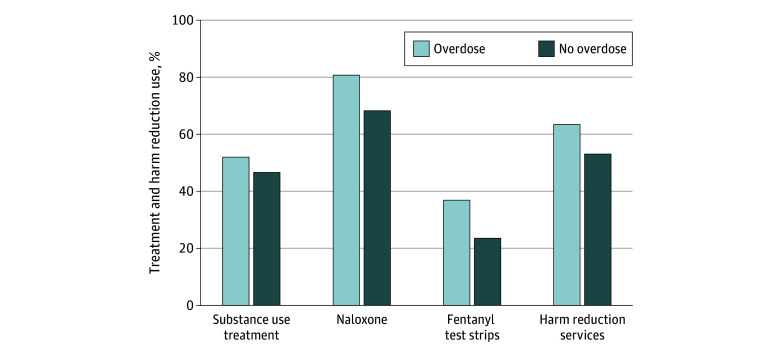
Comparing Treatment and Harm Reduction Use in Overdose vs Nonoverdose Survivors Results of 4 separate logistic regression analyses with the outcomes. Respondents with missing gender, race and ethnicity, age, or overdose experience (n = 18) were dropped from regression analyses. Of the remaining respondents, 2 individuals were dropped from the naloxone model and 1 from the fentanyl test strips model for having missing outcome responses. Predicted probabilities of outcome among those who had and had not experienced an overdose in the past year, adjusted for age, race and ethnicity, gender, and state, are displayed. There were statistically significant differences between overdose vs no overdose groups at *P* < .05 for naloxone, fentanyl test strips, and harm reduction services.

Experiencing an overdose in the past year was associated with increased naloxone possession (80.7%; 95% CI, 73.7%-87.8% vs 68.2%; 95% CI, 58.8%-77.5%; *P* < .001), past 30-day use of fentanyl test strips (36.8%; 95% CI, 26.4%-47.1% vs 23.5%; 95% CI, 15.6%-31.4%; *P* < .001), and past 30-day use of harm reduction services (63.4%; 95% CI, 54.1%-72.7% vs 53.0%; 95% CI, 39.3%-66.8%; *P* = .003) compared with individuals not experiencing an overdose. Past 30-day use of substance use treatment was higher among individuals experiencing an overdose vs those who did not, but this difference was not statistically significant (52.0%; 95% CI, 40.2%-63.7% vs 46.6%; 95% CI, 34.8%-58.3%; *P* = .24) (Figure 1). Drug type, injection drug use, higher frequency of drug use, housing instability, financial insecurity, and criminal legal involvement were associated with an increased probability of experiencing an overdose in univariate and multivariable models (eTable 2 in Supplement 1).

**Figure 2.**
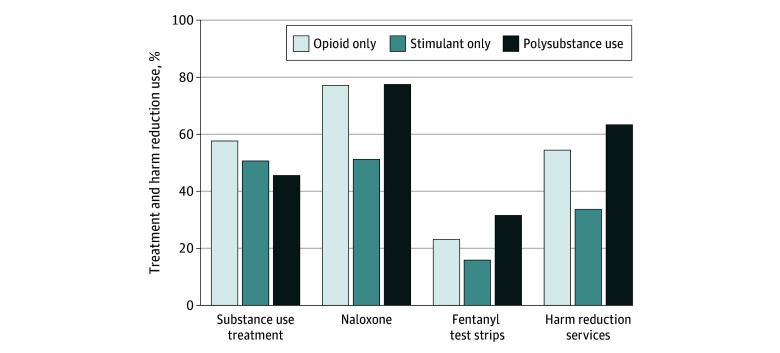
Treatment and Harm Reduction Use by Drug Use Characteristics Results of 4 separate logistic regression analyses with the outcomes. Respondents with missing gender, race and ethnicity, or age (n = 17) were dropped from regression analyses. Of the remaining respondents, 2 individuals were dropped from the naloxone model and 1 from the fentanyl test strips model for having missing outcome responses. Predicted probabilities of outcome by type of drug used in the past 30 days, adjusted for age, race and ethnicity, gender, and state are displayed. Substance use treatment among polysubstance was statistically significantly different at *P* < .05 from opioid only. Naloxone possession among stimulant only was statistically significantly different at *P* < .05 from opioid only and polysubstance. Fentanyl test strip use among stimulant only was statistically significantly different at *P* < .05 from polysubstance. Harm reduction service use among stimulant only was statistically significantly different at *P* < .05 from opioid only and polysubstance.

Treatment and harm reduction practices differed by type of drug use. Substance use treatment was lowest among polysubstance users (45.7%; 95% CI, 34.6%-56.9%), with statistically significant differences compared with opioid-only users (57.8%; 95% CI, 41.6%-74.0%) (*P* = .04) and no significant difference compared with stimulant-only users (50.8%; 95% CI, 35.8%-65.9%) (*P* = .39) (Figure 2). Overall, polysubstance users had higher or similar rates of harm reduction use compared with opioid-only users, and stimulant-only users were less likely to engage in harm reduction practices. Only 51.4% (95% CI, 40.8%-61.9%) of stimulant-only users had naloxone compared with 77.3% (95% CI, 67.4%-87.2%) of opioid-only users (*P* < .001) and 77.6% (95% CI, 69.8%-85.3%) of polysubstance users (*P* < .001). Polysubstance users had the highest rates of fentanyl test strip use (31.7%; 95% CI, 23.0%-40.4%) compared with stimulant-only users (16.0%; 95% CI, 8.4%-23.6%) (*P* < .001) and had higher but not statistically significant differences compared with opioid-only users (23.2%; 95% CI, 12.6%-33.9%) (*P* = .08). Stimulant-only users reported a lower prevalence of harm reduction services use (33.8%; 95% CI, 18.3%-49.2%) compared with polysubstance users (63.5%; 95% CI, 52.6%-74.4%) (*P* < .001) and opioid-only users (54.6%; 95% CI, 37.5%-71.8%) (*P* = .004). State-specific results are reported in eTable 3 and eTable 4 in Supplement 1.

Injection drug use and higher drug use frequency were associated with a lower use of substance use treatment and a higher likelihood of possessing naloxone or fentanyl test strips, and using harm reduction services (eFigure 1 and eFigure 2 in Supplement 1). There were no statistically significant differences in treatment and harm reduction practices among people who differed by housing instability, financial insecurity, and criminal legal involvement measures (eFigures 3-5 in Supplement 1), except for increased fentanyl test strip use among individuals who experienced housing instability vs those who did not (30.7% vs 23.3%; *P* < .001) and increased substance use disorder treatment among individuals with vs without criminal legal involvement (54.8% vs 45.3%; *P* = .04).

Among people who did not receive substance use disorder treatment in the past 30 days, the highest reported barrier was not being ready (32.9%), cost (14.4%), transportation issues (14.1%), not having health insurance (12.3%), and being worried what people would think if they sought treatment (10.8%) (Table 2). Among people who did not receive harm reduction services in the past 30 days, the highest reported barrier was not feeling like they needed services (49.6%), not knowing about services (31.8%), being worried about what people would think if they sought services (11.0%), transportation issues (10.4%), and worried about getting arrested if they sought services (6.7%). Among people who did not use fentanyl test strips in the past 30 days, the highest reported barrier was not knowing what test strips are (33.6%), not wanting to use test strips (29.3%), not having any test strips (16.1%), not knowing where to get test strips (8.7%), and not minding using fentanyl (8.4%).

**Table 2.** Barriers to Substance Use Disorder Treatment, Harm Reduction Service, and Fentanyl Test Strip Use

Barrier^a^	Participants, No. (%)
Among people with no past 30-d treatment use (n = 334)	
Not ready	110 (32.9)
Cost	48 (14.4)
Transportation issues	47 (14.1)
No health insurance	41 (12.3)
Worried what people will think	36 (10.8)
No availability at treatment site	34 (10.2)
Worried about withdrawal	24 (7.2)
Need more information about treatment	20 (6.0)
Worried will be treated poorly	14 (4.2)
Inconvenient locations	13 (3.9)
Too many rules	12 (3.6)
Inconvenient hours	6 (1.8)
Site did not accommodate health care needs	6 (1.8)
Childcare issues	4 (1.2)
Other reason	77 (23.1)
Among people with no past 30-d harm reduction service use (n = 556)	
Do not need services	276 (49.6)
Do not know about services	177 (31.8)
Worried what people will think	61 (11.0)
Transportation issues	58 (10.4)
Worried about getting arrested	37 (6.7)
Worried will be treated poorly	36 (6.5)
Inconvenient hours	26 (4.7)
Other reason	100 (18.0)
Among people with no past 30-d fentanyl test strip use (n = 901)	
Do not know what test strips are	303 (33.6)
Do not want to use test strips	264 (29.3)
Do not have any test strips	145 (16.1)
Do not know where to get test strips	78 (8.7)
Do not mind using fentanyl	76 (8.4)
Hard to get test strips	36 (4.0)
Testing takes too long	7 (0.8)
Worried what people will think	2 (0.2)
Other reason	69 (7.7)

^a^ Categories were not mutually exclusive.

## Discussion

In this cross-sectional study, we collected data on a large, multistate population of people who use drugs. The survey population included a substantial representation of Black and Hispanic people and people who use both stimulants and opioids. These groups have been disproportionately affected by the current overdose wave, yet relatively little is known about their access to harm reduction and treatment services. The study found low adoption of fentanyl test strips and differences in the use of treatment and harm reduction programs in these populations. The study also found that, while past-year overdose survivors had higher rates of treatment and harm reduction service use than people who did not overdose, one-fifth of individuals in this group did not possess naloxone, and only about half were engaged in treatment. Given that people who survive overdose have high risk of subsequent fatal overdose,^27,28^ these findings underscore the need to engage this highly vulnerable subgroup.

Self-described barriers to engaging in treatment among respondents provide several insights into opportunities for intervention. For example, approximately one-quarter of people not involved in treatment said that they were not ready to receive treatment, and the following most common reasons pertained to cost, transportation, and lack of health insurance. Mass media campaigns to reduce stigma toward people who use drugs and promote use of harm reduction and treatment services have been an important aspect of the technical assistance the Bloomberg Overdose Prevention Initiative provides in communities where we recruited VOICES participants. However, additional targeted outreach (eg, through street-based teams) may increase timely access to treatment, especially if outreach can address readiness to start treatment, which should be considered a modifiable attribute.^29,30,31^ Increasing access to low-threshold treatments may also better reach populations not ready for traditional treatment models.^32,33^ The issue of cost and insurance warrants further exploration as expanded Medicaid is available in all 3 states (although Wisconsin does not use the Affordable Care Act coverage provision). Low acceptance of Medicaid in treatment programs and benefit restrictions in Medicaid may impede person-centered, timely access to care.^34,35,36^

Barriers to the use of harm reduction services and fentanyl test strips reflect a self-reported lack of awareness and interest. People who use opioids may not perceive that fentanyl test strips are helpful, given the proliferation of fentanyl in the drug supply. However, reports of not knowing about harm reduction and fentanyl test strips underscore the importance of educating new populations about harm reduction services and tailoring messages to address their concerns. These reported barriers also underscore the need to address structural barriers to accessing harm reduction services. For example, the availability of syringe service programs and broader harm reduction services varies by state, with syringe service programs in particular encountering differing legal, policy, and funding barriers that limit program scale.^37,38,39^

A key theme of the VOICES study is a gap in services and engagement for people who consider themselves exclusive stimulant users. While the increase in overdose deaths that involve stimulants is likely to include many people who intentionally combine drugs, there is also a need to reach people who may not perceive themselves to be using drugs adulterated with other substances. People who primarily smoke or snort stimulants (which was more common among stimulant users in our sample) may not perceive the benefit of accessing harm-reduction tools focused on opioid-related risk factors (naloxone and fentanyl test strips) or may not perceive that they can access safer use supplies other than syringes.^40^ Public health campaigns have recently begun targeting these populations. Still, the scope and breadth of these campaigns remain limited compared with campaigns explicitly focused on people who inject drugs or intend to use opioids.

### Limitations

This study has several limitations. First, the site-based sampling strategy may bias results toward service-engaged respondents. Even though 8.0% of the sample comes from peer recruitment, obtaining responses from individuals wholly removed from all social, harm reduction, and treatment services was still challenging. Second, as self-reported survey responses, our data are subject to social desirability and recall bias. However, this was mediated by having data collectors with extensive training and experience discussing drug use and designing the survey using nonjudgmental language with oversight from a community advisory board. Third, telephone surveys may have excluded people who were unwilling or unable to participate by telephone. However, we provided cell phones to sites to allow clients to call the study telephone line to reduce the impact of this limitation. Fourth, the study was not a probability-based sample; results therefore may not generalize to the broader populations of Michigan, New Jersey, Wisconsin, or other states as local drug markets and service contexts differ greatly. Fifth, only 18 individuals in the sample identified as non-Hispanic Native American or Alaska Native in this study, a sample too small to examine treatment, harm reduction service use, and overdose. Native American populations are experiencing substantially increased rates of overdose death and should be a key recruitment group for similar studies in the future.

## Conclusion

The VOICES study represents one of the most important efforts to characterize the use of treatment and harm reduction services among a racially and ethnically and socially diverse multistate group of people at high risk for drug overdose. The primary finding in our analyses of VOICES survey response data is that access to treatment and harm reduction services is a significant challenge among people who use drugs, especially for people who use stimulants without intentionally using opioids. To increase service use, programs that address gaps in awareness of harm reduction and its benefits and increase the readiness of people to use treatment services will be needed. Amid a rapidly evolving crisis, improving surveillance efforts can provide timely data points to target vulnerable populations for risk-reduction interventions.

## Notes

Supplement 1. **eTable 1.** Site Characteristics (n = 39) **eTable 2.** Associations of Demographics, Structural Vulnerabilities and Drug Use Characteristics With Overdose in the Past Year **eTable 3.** Comparing Treatment and Harm Reduction Use in Overdose Survivors vs Non-Overdose Survivors by State **eTable 4.** Treatment and Harm Reduction Use by Drug Use Characteristics by State **eFigure 1.** Treatment and Harm Reduction Use by Injection Drug Use Status **eFigure 2.** Treatment and Harm Reduction Use by Drug Use Frequency **eFigure 3.** Treatment and Harm Reduction Use by Housing Instability **eFigure 4.** Treatment and Harm Reduction Use by Financial Insecurity **eFigure 5.** Treatment and Harm Reduction Use by Criminal Legal Status **eMethods.** VOICES Survey Instrument

Supplement 2.
Data Sharing Statement
