# Supplementary material for: Harm Reduction and Treatment Among People at High Risk of Overdose
Source: JAMA Netw Open. 2024 Aug 12;7(8):e2427241. doi: 10.1001/jamanetworkopen.2024.27241 (PMC11320172; doi:10.1001/jamanetworkopen.2024.27241)
Supplement: Supplement 2. — Data Sharing Statement [file jamanetwopen-e2427241-s002.pdf]

Data Sharing Statement

Bandara. Harm Reduction and Treatment Among People at High Risk of Overdose. *JAMA Netw Open*. Published August 12, 2024. doi:10.1001/jamanetworkopen.2024.27241

Data
Data available: Yes
Data types: Data dictionary
How to access data: Contact Brendan Saloner, bsaloner@jhu.edu
When available: With publication

Supporting Documents
Document types: Statistical/analytic code, Informed consent form
How to access documents: Contact Brendan Saloner, bsaloner@jhu.edu
When available: With publication

Additional Information
Who can access the data: Researchers whose proposed use of the data has been approved.
Types of analyses: For any purpose
Mechanisms of data availability: After approval of a proposal
